# Plasma levels of D-dimer and fibrin degradation product are unreliable for diagnosing periprosthetic joint infection in patients undergoing re-revision arthroplasty

**DOI:** 10.1186/s13018-021-02764-0

**Published:** 2021-10-19

**Authors:** Hong Xu, Jinwei Xie, Duan Wang, Qiang Huang, Zeyu Huang, Zongke Zhou

**Affiliations:** Department of Orthopaedic Surgery, West China Hospital, Sichuan University, No.37, Guoxue Road, Wuhou District, Chengdu, 610041 Sichuan China

**Keywords:** Re-revision arthroplasty, Periprosthetic joint infection, Diagnosis, Plasma, D-dimer, Fibrin degradation product

## Abstract

**Background:**

The preoperative diagnosis of periprosthetic joint infection (PJI) in patients undergoing re-revision arthroplasty is crucial, so we evaluated whether plasma levels of D-dimer and fibrin degradation product (FDP) could aid such diagnosis.

**Methods:**

We retrospectively analyzed data on patients who underwent re-revision hip or knee arthroplasty at our institute during 2008–2020. Patients were stratified into those who experienced PJI or not, based on 2013 International Consensus Meeting Criteria. Plasma levels of D-dimer and FDP as well as levels of the traditional inflammatory biomarkers C-reactive protein (CRP), erythrocyte sedimentation rate (ESR) and interleukin-6 were compared between the groups. The ability of these biomarkers to diagnose PJI was assessed based on the area under the receiver operating characteristic (AUC) curve, for which predictive cut-offs were optimized based on the Youden index.

**Results:**

Based on a cut-off of 0.80 mg/L, D-dimer gave an AUC of 0.595, high sensitivity of 85.7% but poor specificity of 47.8%. Based on a cut-off of 2.80 mg/L, FDP gave an AUC of 0.550, poor sensitivity of 56.5% and poor specificity of 52.9%. CRP, ESR and interleukin-6 showed much better diagnostic ability, with AUCs > 0.82. The combination of CRP and interleukin-6 gave an AUC of 0.877, high sensitivity of 91.7% and acceptable specificity of 78.3%.

**Conclusions:**

Plasma levels of D-dimer and FDP may be inappropriate for diagnosing PJI in patients undergoing re-revision arthroplasty, whereas the combination of serum CRP and interleukin-6 may be effective.

## Introduction

Total hip arthroplasty (THA) and total knee arthroplasty (TKA) are effective treatments for end-stage hip and knee diseases. Revision-free survival rates are higher than 80% at 25 years after primary TKA [1], or nearly 90% at 15 years after primary THA [2]. Nevertheless, as the number of primary arthroplasties increase, so does the number of revision arthroplasties [3], and a substantial proportion of patients undergoing revision arthroplasty are young [4, 5]. In the US alone, as many as 268,200 revision knee arthroplasties and 97,700 revision hip arthroplasties are expected to be performed in 2030 [6]. This constitutes a substantial medical and economic burden on society and the healthcare system [7].

Another problem with revision arthroplasty is that it can fail, leading to the need for re-revision surgery. The first revision procedure can fail for various reasons, such as aseptic loosening, nonunion, periprosthetic fracture or recurrent dislocation [8, 9]. The most frequent reason for failure, however, is periprosthetic joint infection (PJI) [8], which is associated with high morbidity and mortality. Therefore, screening for PJI before re-revision is crucial for optimizing surgical treatment, protecting the prosthesis and lower limbs, and managing patient expectations [10, 11].

PJI can be diagnosed and pathogens could be isolated based on culture tests of synovial fluid aspirated from the affected joint. Much more convenient, rapid and safe, though, is assay of blood biomarkers of infection [12, 13]. Besides the traditional inflammatory biomarkers, serum CRP and ESR, the 2018 International Consensus Meeting (ICM) Criteria on PJI proposed using serum levels of D-dimer alongside CRP for diagnosing PJI [14], although the usefulness of D-dimer levels is controversial [15, 16]. Another potential biomarker of PJI is fibrin degradation product (FDP), which has been used together with D-dimer to detect fibrinolysis after surgery and to exclude venous thromboembolism [17]. Fujimoto et al. [18] reported that elevated FDP levels stop the negative conversion of serum CRP levels after TKA. Whether plasma D-dimer and FDP are useful for diagnosing PJI in patients undergoing re-revision arthroplasty is unclear. Therefore, we performed a retrospective study to assess their diagnostic value, especially in comparison with the traditional inflammatory biomarkers CRP, ESR and interleukin-6. We also explored whether these biomarkers perform better in combination than on their own.

## Methods

### Study design

This was a single-center retrospective study, which was approved by the Institutional Review Board of our institute (2020-1004) and registered in the Chinese Clinical Trial Registry (ChiCTR2000039989). The Institutional Review Board waived the requirement for written informed consent because it was a retrospective study that had no adverse effects on the health of the included patients, and their information were anonymized during analysis and in the report.

### Patients

We screened all patients who underwent re-revision knee or hip arthroplasty at our institute during 2008–2020 as a result of PJI or aseptic failure. These patients were identified using procedure codes introduced by the 10th Revision of the International Classification of Diseases (Clinical Modification) [19]. We excluded patients who underwent reimplantation surgery because in these cases, the source of pathogens was uncertain [20] and this procedure is part of two-stage arthroplasty in the presence of PJI. We also excluded patients diagnosed with periprosthetic fracture or dislocation, because trauma-induced inflammation and fibrinolysis can significantly affect levels of the biomarkers in our study [21, 22]. Finally, we excluded patients followed up for less than 1 year.

### Diagnosis of PJI

We classified patients into a PJI or non-PJI group according to the 2013 ICM Criteria on PJI [23]. Non-PJI patients had been diagnosed with aseptic failure. We included only non-PJI patients who were followed up for at least 1 year in the clinic or by telephone in order to avoid missing infected cases.

### Laboratory tests

All patients were tested for serum levels of CRP and ESR, reflecting routine practice at our hospital. Most of the patients admitted from 2012 onwards were tested for plasma D-dimer, and most of them admitted from 2015 onwards were also tested for plasma FDP [24]. Most of the patients in our cohort were tested for interleukin-6. And these biomarkers were tested preoperatively.

If a patient had suspected PJI based on medical history, especially a diagnosis of after the first revision arthroplasty, the levels of serum CRP and ESR, as well as subjected to physical and x-ray examinations, the involved joint had been aspirated. In addition, the aspiration was performed, either by experienced technicians from the Department of Doppler Ultrasonography in the case of hip joints, or by surgeons in a specialized aspiration room in the case of knee joints. All aspirations were performed with strict attention to sterile procedure.

The obtained synovial fluid was immediately sent to the Department of Laboratory Medicine at our institute for culture and/or other tests. Aerobic and anaerobic cultures were prepared and maintained for 5 days routinely, and white blood cell counts, neutrophil differential counts, and polymorphonuclear neutrophil percentages were determined. If a limited volume of aspirated synovial fluid was available, only culture tests were performed. In addition, synovial fluid was sent for culturing tuberculosis only if the patient was highly suspected of tuberculosis infection, which maintained for 42 days.

In addition, four or more soft tissue locations around the implant were collected and sent for culture tests and histology analyses. We defined the positive histology as: > 5 neutrophils per high-power field in 5 high-power fields (× 400) according to the 2013 ICM Criteria on PJI [23].

### Outcomes

The outcomes of this study were the following determinations: serum levels of CRP and interleukin-6; ESR; plasma levels of D-dimer and FDP before re-revision arthroplasty; and final diagnosis of PJI or aseptic failure based on 2013 ICM Criteria on PJI [23]. Significantly, the patients with some comorbidities, which may affect the levels of inflammatory or fibrinolytic biomarkers, such as chronic obstructive pulmonary disease, coronary heart disease, rheumatoid arthritis and psoriasis, were included in our analyses due to the confounding effects and the limitation of sample size [25].

### Statistical analyses

Normally distributed continuous data were reported as the mean and standard deviation (SD), and inter-group differences were assessed for significance using Student’s *t* test. Continuous data with a skewed distribution or unequal variance were reported as the median and interquartile range (IQR), and differences were assessed using the Wilcoxon Mann–Whitney *U* test. Categorical variables were reported as frequency and percentages, and differences were assessed using Pearson’s chi-squared test or Fisher’s exact test. Differences associated with *P* < 0.05 were considered statistically significant.

When measured values of biomarkers fell below the manufacturer-specified limit of detection, the values were reported as that limit. For example, plasma levels of FDP < 2.5 mg/L were reported as 2.5 mg/L, while serum levels of IL-6 < 1.5 pg/ml were reported as 1.5 pg/ml.

Receiver operating characteristic curves were generated to examine relationships between the true-positive rate (sensitivity) and false-positive rate (1-specificity) of the various biomarkers, and their optimal predictive cut-offs were determined according to the Youden index. The diagnostic ability of these biomarkers was also compared in terms of the area under the curves (AUC). The cut-offs recommended by the 2013 ICM Criteria on PJI [23] were also applied in the case of CRP (10 mg/L) and ESR (30 mm/h). Positive predictive value (PPV) and negative predictive value (NPV) were also evaluated.

## Results

**Fig. 1:**
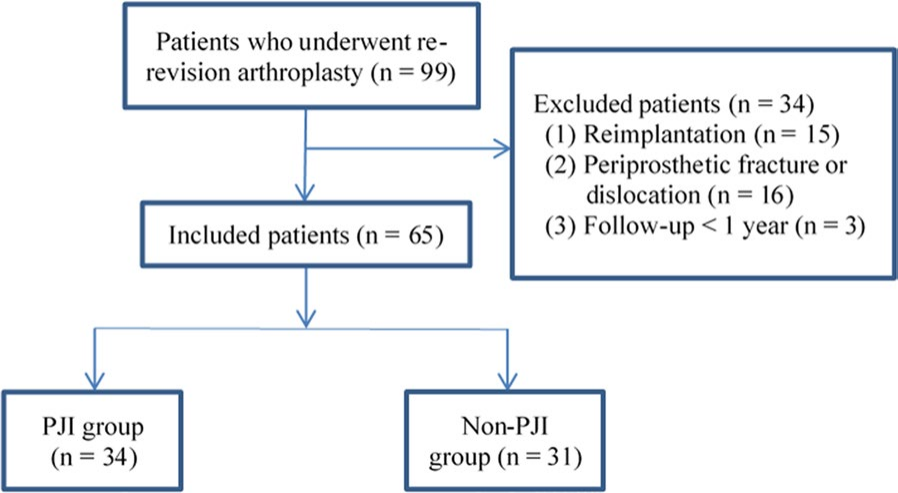
Flowchart of patient enrollment

We initially enrolled 99 patients but then excluded those who underwent reimplantation surgery (*n* = 15), who were diagnosed with periprosthetic fracture or dislocation (*n* = 16), and those who were followed up for less than one year (*n* = 3). In the end, 65 patients were analyzed, comprising 34 with PJI and 31 with aseptic failure (non-PJI) (Fig. 1).

Serum levels of CRP and ESR were assayed in all patients, while levels of interleukin-6 were assayed in 24 PJI patients and 23 non-PJI patients. Plasma levels of D-dimer were assayed in 28 PJI patients and 23 non-PJI patients, while plasma levels of FDP were assayed in 23 PJI patients and 17 non-PJI patients. PJI patients contained significantly higher levels of CRP, ESR and interleukin-6. In contrast, the two groups of patients did not differ significantly in D-dimer [1.35 (0.89–2.18) vs 0.86 (0.62–2.37), *P* = 0.248], FDP [3.20 (2.50–6.00) vs 2.50 (2.50–5.25), *P* = 0.248] (Table 1).

**Table 1:** Biomarkers in patients diagnosed with PJI or not who underwent re-revision knee or hip arthroplasty

Biomarker	PJI group		Non-PJI group		*P* value
	*n*	Median (IQR)	*n*	Median (IQR)	
CRP (mg/L)	34	25.40 (16.61–60.68)	31	3.11 (1.77–7.71)	< 0.001^*^
ESR (mm/h)	34	70.11 (53.50–80.00)	31	25.00 (13.00–45.00)	< 0.001^*^
D-dimer (mg/L)	28	1.35 (0.89–2.18)	23	0.86 (0.62–2.37)	0.248
FDP (mg/L)	23	3.20 (2.50–6.00)	17	2.50 (2.50–5.25)	0.576
IL-6 (pg/L)	24	25.45 (6.66–67.33)	23	2.67 (1.76–9.54)	< 0.001^*^

n, sample size. CRP, C-reactive protein; ESR, erythrocyte sedimentation rate; FDP, fibrin degradation product; IL-6, interleukin-6; IQR, interquartile range; PJI, periprosthetic joint infection. ^*^: *P* < 0.05

**Fig. 2:**
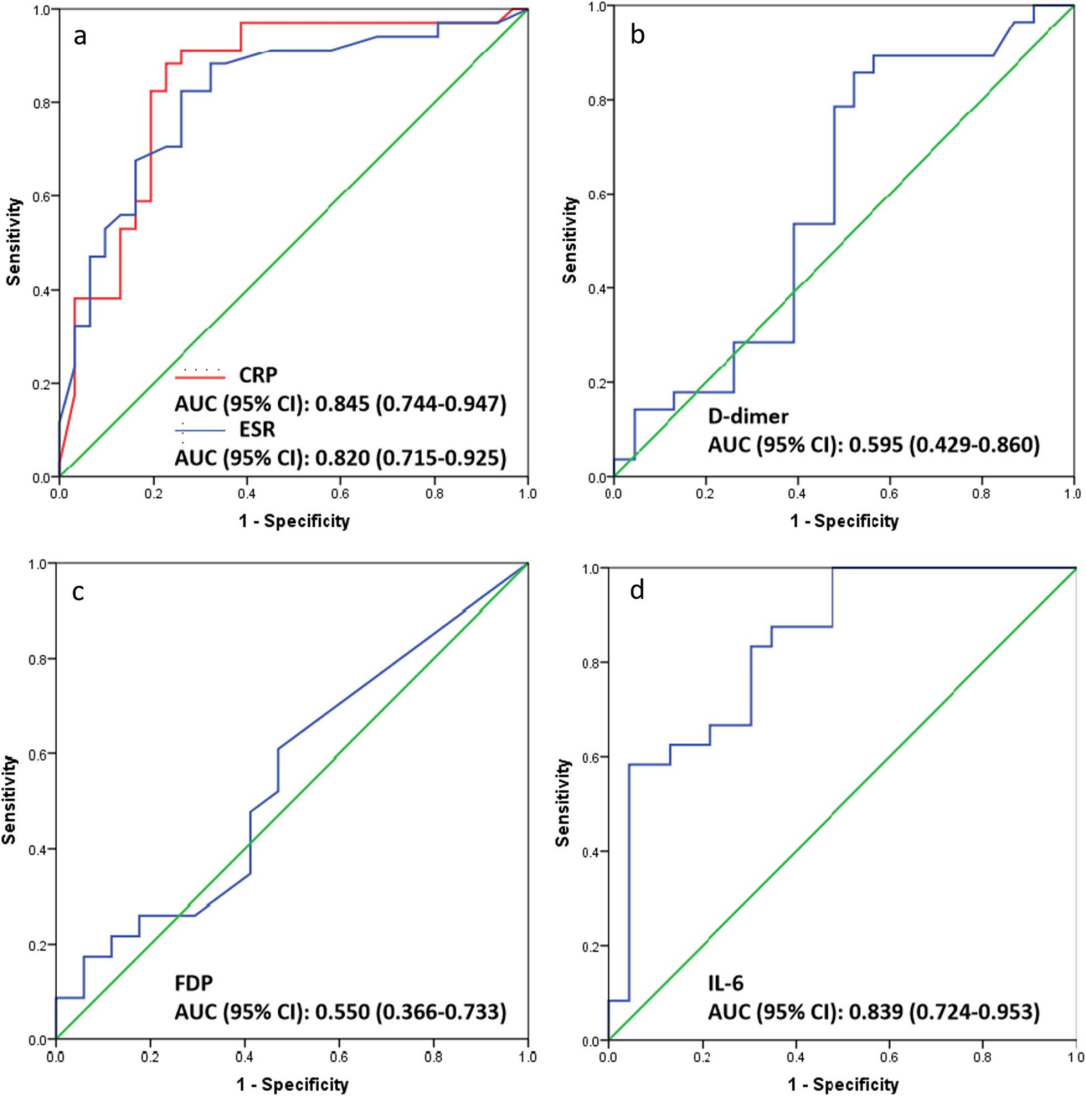
Receiver operating characteristic curves of individual biomarkers. **a** Serum level of C-reactive protein (CRP) and erythrocyte sedimentation rate (ESR). **b** Plasma level of D-dimer. **c** Plasma level of fibrin degradation product (FDP). **d** Serum level of interleukin-6 (IL-6)

Next, we evaluated the ability of each of the biomarkers to identify PJI in patients undergoing re-revision arthroplasty (Table 2). CRP showed the highest AUC (0.845, 95% CI 0.744–0.947), followed by interleukin-6 (0.839, 95% CI 0.724–0.953) and ESR (0.820, 95% CI 0.715–0.925) (Fig. 2a, b). CRP showed a high sensitivity of 82.4% and acceptable specificity of 77.2% at the recommended cut-off of 10 mg/L [23], while its sensitivity increased to 88.2% at the optimal cut-off of 8.40 mg/L derived from the Youden index in the present study. ESR showed a high sensitivity of 82.4% and low specificity of 67.7% at the recommended cut-off of 30 mm/h [23], and its specificity increased to 74.2% with the optimal cut-off of 30 mm/h derived from the Youden index. Interleukin-6 gave a high sensitivity of 83.3% but low specificity of 69.6% with the optimal cut-off of 6.00 pg/L derived from the Youden index.

**Table 2:** Diagnostic performance of biomarkers individually

Biomarker	AUC (95% CI)	Youden index	Cut-off	Sensitivity (%)	Specificity (%)	PPV (%)	NPV (%)
CRP (mg/L)	–	0.598	10.00*	82.4	77.4	80.0	80.0
	0.845 (0.744–0.947)	0.656	8.40^#^	88.2	77.4	81.1	85.7
ESR (mm/h)	–	0.501	30.00*	82.4	67.7	73.7	77.8
	0.820 (0.715–0.925)	0.566	33.00^#^	82.4	74.2	77.8	79.4
D-dimer (mg/L)	0.595 (0.429–0.860)	0.335	0.80	85.7	47.8	66.7	73.3
FDP (mg/L)	0.550 (0.366–0.733)	0.094	2.80	56.5	52.9	61.9	47.3
IL-6 (pg/L)	0.839 (0.724–0.953)	0.529	6.00	83.3	69.6	74.1	80.0

AUC, area under the receiver operating characteristic curve; CI, confidence interval; CRP, C-reactive protein; ESR, erythrocyte sedimentation rate; FDP, fibrin degradation product; IL-6, interleukin-6; PPV, positive predictive value; NPV, negative predictive value

^*^From the 2013 International Consensus Meeting Criteria on PJI [23]

^#^Based on the Youden index for our cohort

D-dimer and FDP performed substantially worse at diagnosing PJI. Both gave lower AUCs: (0.595, 95% CI 0.429–0.860) for D-dimer and (0.550, 95% CI 0.366–0.733) for FDP, respectively. D-dimer showed a high sensitivity of 85.7% but unacceptable specificity of 47.8% with the optimal cut-off of 0.80 mg/L derived based on the Youden index (Fig. 2c). FDP gave poor sensitivity of 56.5% and poor specificity of 52.9% with the optimal cut-off of 2.80 mg/L based on the Youden index (Fig. 2d).

**Fig. 3:**
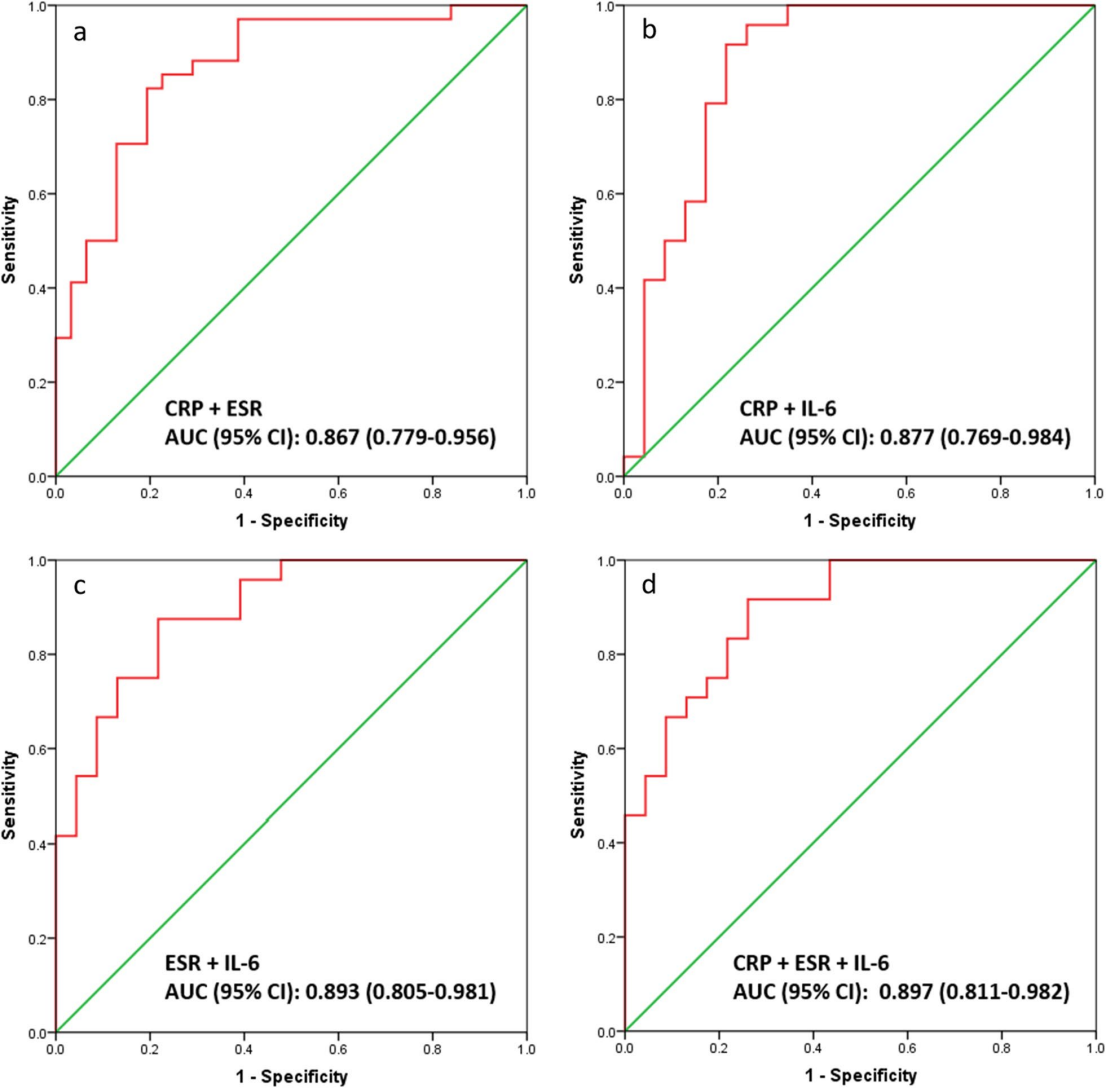
Receiver operating characteristic curves of biomarkers in combination. **a** Combination of C-reactive protein (CRP) and erythrocyte sedimentation rate (ESR). **b** Combination of CRP and interleukin-6 (IL-6). **c** Combination of ESR and IL-6. **d** Combination of CRP, ESR and IL-6

Finally, we evaluated the ability of different combinations of CRP, ESR and IL-6 to diagnose PJI in our cohort (Table 3, Fig. 3). The combination of CRP and interleukin-6 gave a high sensitivity of 91.7% and acceptable specificity of 78.3%, with an AUC of (0.877, 95% CI 0.769–0.984). It also gave acceptable PPV and the highest NPV (90.0%) of all combinations. None of the other combinations performed better.

**Table 3:** Diagnostic performance of biomarkers in combination

Combination	AUC (95% CI)	Youden index	Sensitivity (%)	Specificity (%)	PPV (%)	NPV (%)
*Combination of two markers*						
CRP + ESR	0.867 (0.779–0.956)	0.630	82.4	80.6	82.3	80.7
CRP + IL-6	0.877 (0.769–0.984)	0.700	91.7	78.3	81.5	90.0
ESR + IL-6	0.893 (0.805–0.981)	0.658	87.5	78.3	80.8	85.75
*Combination of three markers*						
CRP + ESR + IL-6	0.897 (0.811–0.982)	0.656	91.7	73.9	78.6	89.5

AUC, area under the receiver operating characteristic curve; CI, confidence interval; CRP, C-reactive protein; ESR, erythrocyte sedimentation rate; IL-6, interleukin-6; PPV, positive predictive value; NPV, negative predictive value

## Discussion

This appears to be the first study evaluating the diagnostic value of plasma levels of D-dimer and FDP for identifying PJI in patients undergoing re-revision hip or knee arthroplasty. Our results suggest that neither biomarker is useful for this purpose. Instead, we identified the combination of serum levels of CRP and interleukin-6 for screening PJI with high sensitivity and acceptable specificity in these patients.

Re-revision arthroplasty is more challenging than the first revision procedure because of the complexity of the surgery; the generally poor condition of patients, who may have lost bone mass or suffered soft-tissue scarring; and the high expectations of the patients. Failure to screen for PJI before re-revision arthroplasty increases the risk of postoperative recurrence of infection. Therefore researchers have explored various biomarkers that can easily be assayed in order to screen patients for PJI [26]. D-dimer is widely assayed to exclude venous thromboembolism [27], and its levels are strongly associated with inflammation [28] and infection [29]. Shahi et al. [30] reported that serum D-dimer levels could diagnose PJI with even greater sensitivity (89%) and specificity (93%) than CRP and ESR, and on the basis of that work, the ICM proposed serum D-dimer alongside CRP as a way to diagnose PJI in 2018 [14]. However, several studies reported divergent results about the diagnostic reliability of D-dimer [16, 31–33], perhaps in part because some studies examined levels in serum [30, 32, 33], while others assayed it in plasma [24, 34]. In fact, we and others [35] believe that some of those studies did not adequately differentiate between assays performed in serum or plasma. For this reason, meta-analyses of the diagnostic ability of D-dimer may be also misleading, though most of them seem to agree that serum levels have greater diagnostic value than plasma levels [15, 36, 37]. To help resolve this issue, we are performing a prospective, parallel comparison of plasma- and serum-based assays of D-dimer for identifying PJI before revision arthroplasty, we believe that its results may allow definitive conclusions about the diagnostic value of plasma/serum D-dimer [38].

FDP refers to the fragments of fibrin and fibrinogen generated by plasmin, its level are strongly associated with the state of the fibrinolytic system [39]. One study suggested that FDP may be useful for PJI diagnosis [18]. Nevertheless, our small study suggests that FDP is unreliable for diagnosing PJI before re-revision arthroplasty, and a previous study of patients undergoing first revision arthroplasty came to a similar conclusion [24].

Serum CRP and ESR are the classical biomarkers for diagnosing PJI [14]. In our cohort, CRP proved superior to ESR and interleukin-6, based on a cut-off of 8.4 mg/L, which is slightly lower than that recommended by the ICM [14, 23]. However, as one of the first-line indicators for screening PJI, we believe that the relatively low cut-off of CRP may help reduce false-positive diagnoses of PJI. Furthermore, we were able to increase both the sensitivity and specificity of CRP by combining it with interleukin-6. The combination of these two biomarkers therefore shows promise for screening patients for PJI before re-revision arthroplasty. They may be more suitable as a first-line screening tool than synovial fluid tests, which may be impractical because of “dry joint” in some cases [40], or dangerous because of contamination of the aspirated joint [41]. Furthermore, joint aspiration requires technical skill and suitable equipment, it may be inconvenient, especially for the hip joints and outpatients. By contrast, blood biomarkers are convenient, fast and safe, which are irreplaceable for the preliminary identification of PJI.

There are some limitations of our study that should be taken into consideration when interpreting our findings. First, this is a retrospective study and biomarker data were missing for some patients. Nevertheless, the data of our cohort were adequate for demonstrating that plasma levels of D-dimer and FDP were unreliable for diagnosing PJI in patients before re-revision arthroplasty. Second, our sample was small, so our findings need to be verified and extended in larger studies. Indeed, the small sample prevented us from evaluating the effect of comorbidities, such as embolism and rheumatoid arthritis, on the diagnostic value of the tested biomarkers.

## Conclusion

Plasma levels of D-dimer and FDP are unreliable for diagnosing PJI in patients undergoing re-revision arthroplasty. In contrast, the combination of serum levels of CRP and interleukin-6 may be promising for screening PJI in these patients. Larger studies are needed to validate and extend our findings.

## Data Availability

Please contact author for data requests.

## Abbreviations

AUC: Area under the receiver operating characteristic
CRP: C-reactive protein
ESR: Erythrocyte sedimentation rate
FDP: Fibrin degradation product
ICM: International Consensus Meeting
IQR: Interquartile range
NPV: Negative predictive value
PJI: Periprosthetic joint infection
PPV: Positive predictive value
SD: Standard deviation
THA: Total hip arthroplasty
TKA: Total knee arthroplasty
